# Reactive Nitrogen Hotspots Related to Microscale Heterogeneity in Biological Soil Crusts

**DOI:** 10.1021/acs.est.2c02207

**Published:** 2022-08-05

**Authors:** Alexandra Maria Kratz, Stefanie Maier, Jens Weber, Minsu Kim, Giacomo Mele, Laura Gargiulo, Anna Lena Leifke, Maria Prass, Raeid M. M. Abed, Yafang Cheng, Hang Su, Ulrich Pöschl, Bettina Weber

**Affiliations:** † Multiphase Chemistry Department, Max Planck Institute for Chemistry, Mainz 55128, Germany; ‡ Institute of Biology, Division of Plant Sciences, University of Graz, Graz 8010, Austria; § Institute for Agriculture and Forestry in the Mediterranean, National Council of Research, 80055 Portici, Italy; ∥ College of Science, Biology Department, Sultan Qaboos University, P.O. Box 36, Al Khoud, Seeb 123, Sultanate of Oman

**Keywords:** biological soil crusts, reactive nitrogen, nitrous acid (HONO), nitric oxide (NO), microsensors, X-ray microtomography, fluorescence microscopy

## Abstract

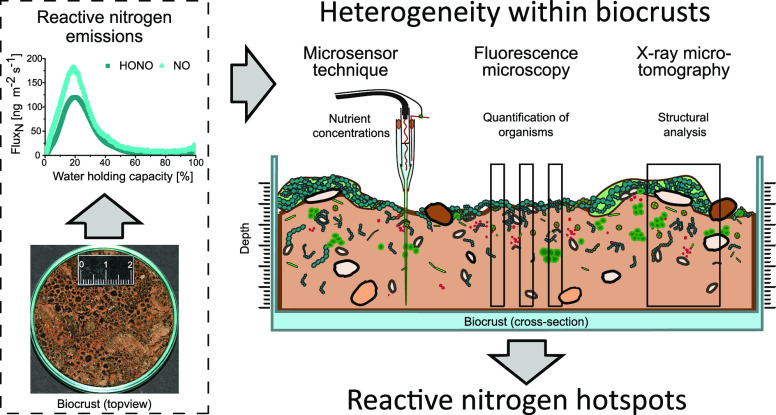

Biocrusts covering drylands account for major fractions of terrestrial biological nitrogen fixation and release large amounts of gaseous reactive nitrogen (N_r_) as nitrous acid (HONO) and nitric oxide (NO). Recent investigations suggested that aerobic and anaerobic microbial nitrogen transformations occur simultaneously upon desiccation of biocrusts, but the spatio-temporal distribution of seemingly contradictory processes remained unclear. Here, we explore small-scale gradients in chemical concentrations related to structural characteristics and organism distribution. X-ray microtomography and fluorescence microscopy revealed mixed pore size structures, where photoautotrophs and cyanobacterial polysaccharides clustered irregularly in the uppermost millimeter. Microsensor measurements showed strong gradients of pH, oxygen, and nitrite, nitrate, and ammonium ion concentrations at micrometer scales in both vertical and lateral directions. Initial oxygen saturation was mostly low (∼30%) at full water holding capacity, suggesting widely anoxic conditions, and increased rapidly upon desiccation. Nitrite concentrations (∼6 to 800 μM) and pH values (∼6.5 to 9.5) were highest around 70% WHC. During further desiccation they decreased, while emissions of HONO and NO increased, reaching maximum values around 20% WHC. Our results illustrate simultaneous, spatially separated aerobic and anaerobic nitrogen transformations, which are critical for N_r_ emissions, but might be impacted by future global change and land management.

## Abstract 2

Reactive nitrogen emissions of biocrusts are linked to microscale processes driven by microorganisms heterogeneously colonizing substrates of mixed pore sizes.

## Introduction

Biological soil crusts (biocrusts) are assemblages of lichens, bryophytes, and microbes that colonize the uppermost layer of soil in dryland ecosystems and cover ∼12% of the global terrestrial surface.^1−5^ About 40% of the global terrestrial biological N fixation have been attributed to biocrusts,^6^ sustaining soil fertility in nutrient-deprived dryland ecosystems.^7−9^ Some of the fixed nitrogen can be re-emitted to the atmosphere in the form of nitrous oxide (N_2_O),^10,11^ ammonia (NH_3_),^12,13^ nitric oxide (NO), and nitrous acid (HONO).^14−23^ The estimated global emissions of reactive nitrogen (N_r_, HONO, and NO) amount to ∼1.7 Tg a^–1^, corresponding to ∼20% of N_r_ from soils under natural vegetation cover.^20,24^

Nitrogen oxides (NO_*x*_ = NO + NO_2_) and HONO are key species in the global N cycling and contribute to the production of tropospheric ozone (O_3,_ short-lived climate pollutant) and hydroxyl radicals (OH^•^), which regulate the oxidizing power and self-cleaning capacity of the atmosphere.^25−31^ N gas emissions from biocrusts, which host a special type of soil microbiome,^32^ and soil may be mainly promoted by the biotic processes N-fixation, nitrification, and denitrification.^7,20,21,23,32−34^ Under aerobic and anaerobic conditions in biocrusts and soil, nitrite (NO_2_^–^) can be formed during nitrification and denitrification, respectively, and can be released as HONO to the atmosphere.^7,20,21,23,32^ Biological sources of NO include NH_3_-oxidizing bacteria (AOB), which mediate the first phase of nitrification and carry out nitrifier denitrification under oxygen-limited conditions.^33,34^

NO and HONO emissions are known to be strongly related to the water content.^35^ In most studies, emissions from drying soil and biocrusts were lowest at high water holding capacity (100% WHC) and reached maximum values around 20–30% WHC.^18,20,21,23^ In another study, emissions of NO and HONO were shown to also occur at high water contents.^36^ In former studies, HONO and NO emissions were analyzed by means of continuous flux measurements from bulk soil,^23^ soil bacteria,^21^ and biocrusts.^18,20,37^ For biocrusts, a high variability in the total flux values was observed.^18,20,32^ A drawback of flux measurements is, however, that they only give the integrated balance of gas exchange at the sample scale (∼cm), whereas they do not allow to draw conclusions at the scale relevant to microorganisms, substrate concentrations at microsites and processes occurring at pore scales. In former studies it has been observed that for example N_2_O emissions occur in hotspots during hot moments, but to our knowledge this has not been described for N_r_ emissions, and the underlying small-scale processes have not been analyzed in detail.^38−40^

One method that allows such small-scale analyses is the application of microsensor techniques. The method of liquid ion-exchange (LIX) based microsensors has been developed to detect and analyze the concentrations of different ions, such as ammonium (NH_4_^+^), NO_2_^–^, nitrate (NO_3_^–^), and H^+^ (pH). They have been used to conduct in-depth analyses of dynamic processes in artificial biofilms (in a porous substrate photobioreactor), as well as in wastewater biofilms and flocs to investigate the sulfate reduction and denitrification.^41,42^ Johnson et al.^43^ utilized LIX microsensors to directly measure the denitrification and nitrogen export in biocrusts to gain information on the fate of the fixed N.

In earlier studies, we observed that various, partly contradictory processes, such as N fixation, nitrification, denitrification, ammonification, and anaerobic ammonium oxidation (anammox), occur at the same time within a single piece of biocrust (see Figure 4c in Maier et al.^32^). Thus, the objective of this study was to understand how spatial heterogeneity within biocrusts and patterns in the local distribution of soil microbiota affect soil processes and hence gas fluxes at larger scales. The specific objectives were to (i) investigate the pore structure, utilizing X-ray microtomography (micro-CT) at different hydration stages, (ii) study the distribution of microbes in the microenvironments by means of fluorescence microscopy, and (iii) analyze the small-scale variation of physicochemical parameters (i.e., pH, O_2_, and NH_4_^+^, NO_3_^–^, and NO_2_^–^) using microsensors under varying water contents. We aimed to address the influence of localized conditions of soil water, pH, NO_2_^–^, NO_3_^–^, and NH_4_^+^ on N_r_ emissions, especially HONO and NO, within a heterogeneous soil environment.

## Material and Methods

### Study Area

Samples for the analyses were collected next to the former BIOTA observatory of Soebatsfontein (30.1865°S, 17.5433°E, 392 m a.s.l), located within the Succulent Karoo biome, 60 km south of Springbok.^44−46^ The Succulent Karoo is a semiarid dryland region, and its biome covers an area of about 103,000 km^2^. This region is considered to be a diversity hotspot of global significance, which is mainly due to a high diversity of succulent plants, with many species being of major conservation importance.^47,48^ At the observatory of Soebatsfontein, the temperature ranges from 3.5 °C (July) to 42.5 °C (February) with a mean air temperature of 19.4 °C.^37,45,46^ The annual precipitation amounts to ∼131 (97–175) mm and most of the precipitation occurs between July and August with ∼45 precipitation events per year.^37,45,46,49^

### Sampling

Cyanobacteria-dominated biocrusts with cyanolichens were collected in March, at the end of the dry season, in small Petri dishes (55 mm diameter and 10 mm height). For sampling, the bottom of the Petri dish was placed upside down on the biocrust surface, pressed into the substrate, and lifted with the help of a trowel pushed below. The samples were turned in an upright position and, in order to minimize metabolic activity, they were air-dried and subsequently transported to the Max Planck Institute for Chemistry (MPIC) in Mainz, Germany, for further analyses. For further details on the identification of species, refer to the Supporting Information, Methods.

### X-Ray Microtomography

Structural analyses (pore size distribution and vertical porosity profiles) were performed with an X-ray microtomograph (micro-CT, Bruker 1272 system) using a scanning protocol with the parameters presented in Table S1A. Image reconstruction was conducted with NRecon software v. 1.7.1 applying the cone-beam algorithm of Feldkamp with the parameters indicated in Table S1B.

The crust sample chosen for the analysis was weighed (initial state), then saturated with distilled water and weighed again (Table S2). The crust structure was analyzed at the two states of ∼50 and 0% water holding capacity (WHC). For further details on the methodology, see the Supplementary Methods.

### Fluorescence Microscopy

Fluorescence microscopy was used to localize and visualize photoautotrophic organisms (algae, cyanobacteria, cyanolichens) and complex polysaccharides [e.g., chitin, cellulose, and EPS (extracellular polymeric substances of cyanobacteria)], the latter indicating fungi and cyanobacterial sheaths in cross-sections of the cyanobacteria-dominated biocrusts. To assess the spatial distribution of photoautotrophic organisms, the autofluorescence of chlorophyll_a_ was used. Complex polysaccharides were visualized using ready to use Calcofluor-White (CFW) stain. During fluorescence microscopy, two distinctive fractions of the biocrust samples were defined as photoautotrophic and heterotrophic layers (PL and HL, respectively) where the PL contains cyanobacteria, lichens, and bryophytes and the HL is devoid of photoautotrophs, but comprises fungi, bacteria, archaea, as well as microfauna (protozoa, nematodes and microarthropods). For further details regarding microscopy, see the Supplementary Methods.

### Microsensor Measurements

Vertical concentration/saturation gradients were investigated during desiccation of biocrusts at 25 °C and in the dark, to be congruent with the conditions during dynamic chamber measurements. LIX sensors with a tip diameter of 20–30 μm were used for the determination of pH, NO_3_^–^, NO_2_^–^, and NH_4_^+^ concentrations and were produced as explained by de Beer et al.,^50−52^ with slight adaptions for measurements in biocrust samples. In order to confer more stability, the sensor tips were thickened and cut with a diamond knife at the requested diameter of about 20–30 μm (inner diameter) under the microscope. pH measurements were obtained with LIX sensors and pH microelectrodes (pH-100, Unisense A/S, Aarhus, Denmark) with a tip diameter of 100 μm. O_2_ saturation was analyzed using oxygen microsensors (OX-100, Unisense A/S, Aarhus, Denmark) with a tip diameter of 100 μm.

For the measurements, biocrust samples were saturated with sterile, artificial rainwater^53,54^ and drained by gravity to achieve full WHC. The measurements were conducted during one desiccation cycle (wetting and subsequent drying). At least 15 vertical profiles were measured in the dark at hourly intervals over the course of desiccation. For each sensor type, at least five replicate measurements were conducted at different locations within biocrust samples. The sensors were moved in a vertical manner by means of a motorized micromanipulator (MM33, Märzhäuser Wetzlar GmbH & Co. KG, Wetzlar, Germany) to a depth of 5 mm. A typical desiccation cycle showed a linear decrease of the water content expressed as percentage of WHC (Figure S1), referring to the whole Petri dish serving as reference.

Statistical differences between the PL and the HL layers were analyzed for the microsensor profiles using the Mann–Whitney U test, since the data were not normally distributed (OriginLab Corporation, Northampton, Massachusetts, USA; Table S4). For more detailed information, see the Supplementary Methods.

### Dynamic Chamber Measurements

The analysis of HONO and NO emissions was carried out in an air-flushed dynamic Teflon chamber.^18,20−23^ HONO was detected spectrophotometrically, using a long path absorption photometer (LOPAP), whereas NO and NO_2_ were analyzed with a gas chemiluminescence detector equipped with a blue light converter.^18,20,21,23^ Measurements occurred at 25 °C in the dark to avoid photochemical reactions, and the samples were fully wetted to 100% WHC, placed in the chamber, and measured until complete desiccation. Further methodological information is provided in the Supplementary Methods.

## Results and Discussion

### X-ray Microtomography

**Figure 1:**
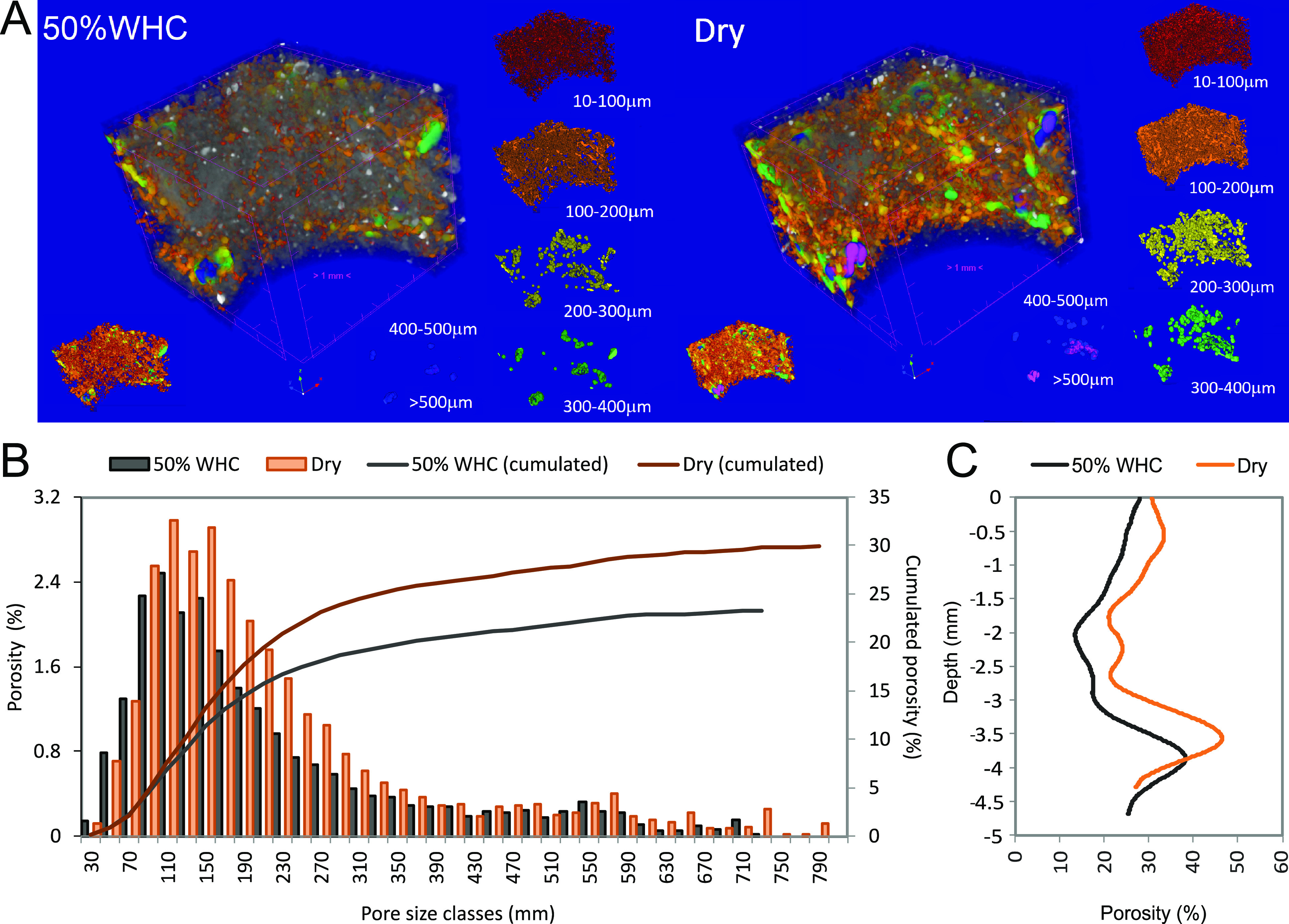
Structure and porosity of one biocrust sample at two hydration states: (A) 3D visualization of the biocrust pores at ∼50% water holding capacity (WHC) corresponding to ∼80% field capacity (FC) (left) and in a dry state (right), with pore volume size (μm) classified by color scale. Solid phase is shown in gray color, white spots are mineral grains inside the soil matrix; (B) pore size distribution at ∼50% WHC and in a dry state; (C) vertical porosity profile of the crust sample at ∼50% WHC and in a dry state.

The pore-size distribution and vertical gradient of porosity of a biocrust sample was investigated at two hydration conditions, ∼50 and 0% WHC, by means of X-ray microtomography (Figures 1 and S2). The obtained images showed that the locations of macropores did not change upon desiccation, whereas the overall volume of the crust sample decreased by 8% (from 1.64 to 1.51 cm^3^; Figures 1A and S2). Total porosity increased from 23.3% at ∼50% WHC to 29.9% in a dry state, maximum pore size diameters rose from 710 to 790 μm, and the mean pore size increased from 212 to 223 μm (Figure 1B). The main changes in pore diameter occurred between 70 and 350 μm (Figure 1B), while the vertical profile of porosity, measured along the entire biocrust sample, showed only minute changes (Figure 1C). Porosity was low between ∼2–3 mm depth (∼22% in a dry and <20% in a wet state), whereas above and below higher porosities were measured (with maximum values of ∼45 and ∼38% porosity in a dry and wet state, respectively). Local vertical profiles of porosity measured in a dry state showed great heterogeneity within the crust sample with the standard deviation of locally measured porosity values ranging from ∼10% in the upper (until 2.2 mm depth) and lower (below 3.7 mm depth) part of the crust to ∼30% between 2.2 and 3.7 mm depth (Figure. S3D). In this central layer, minimum and maximum porosity ranged from 0 to 100% (Figure. S3C), indicating presence of both solid aggregates and macropores. In total, only a general shrinkage of the solid phase occurred, which caused increased pore sizes within the biocrust along with a reduction of its overall volume. Consecutive microsensor profiling also showed a shrinkage of the biocrust, indicated by a retraction of the biocrust surface over the course of desiccation. Similarly, Rodríguez-Caballero et al.^55^ showed an initial swelling of the biocrust surface upon hydration, followed by a shrinking during desiccation. They highlighted that the X-ray microtopography of the biocrust surface changed during hydration, with an increase in surface height and roughness (up to 0.24 and 0.20 mm, respectively) for lichen- and cyanobacteria-dominated biocrusts. There are several studies using X-ray microtomography to investigate the biocrust structure after disturbance,^56^ to compare different ages of biocrusts,^57^ or to show changes of the pore structure during crust succession.^58^ Similar to our investigations on biocrust structure, the study of Couradeau et al.^59^ showed that sand grains stayed in place, despite the fact that the microenvironment of EPS shrank during desiccation. This was mainly because the EPS sheaths did not strongly adhere to the sand grains. They could also show that EPS sheath material remained effectively hydrated while the surrounding soil pore regions were steadily drying. In another study, microbial extracellular polymeric substances (EPS) were described to alter soil water retention by reversible swelling of the cross-linked polymer matrix.^60^

### Fluorescence Microscopy

**Figure 2:**
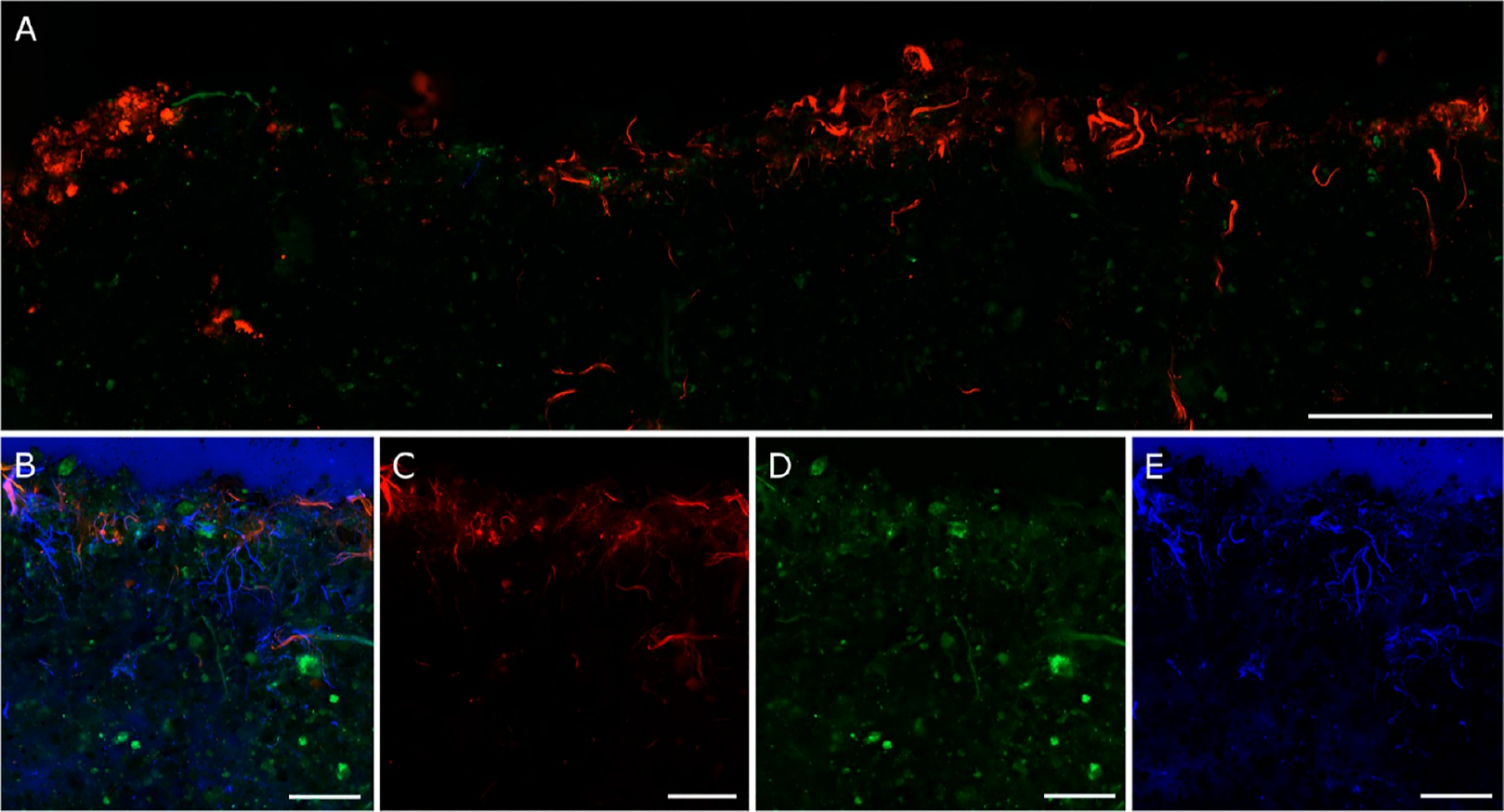
Fluorescence microcopy of biological soil crusts: (A) exemplary illustration of merged fluorescence micrographs of one cross-section of a cyanobacteria-dominated biocrust sample. The red channel shows photoautotrophic organisms; pedological features (e.g., sand grains, stones) are shown in green color. Scale represents 2.5 mm. (B,C,D,E) represent a subsection of a cyanobacteria-dominated biocrust. (B) Merged fluorescence image; (C) red channel, representing photoautotrophic organisms; (D) green channel, showing soil pedological features (e.g., sand grains, stones); and (E) blue channel, showing polysaccharides like chitin, cellulose, and the cyanobacterial extracellular polymeric substances (EPS). Scales represent 600 μm. For additional images, see Figure S4.

**Figure 3:**
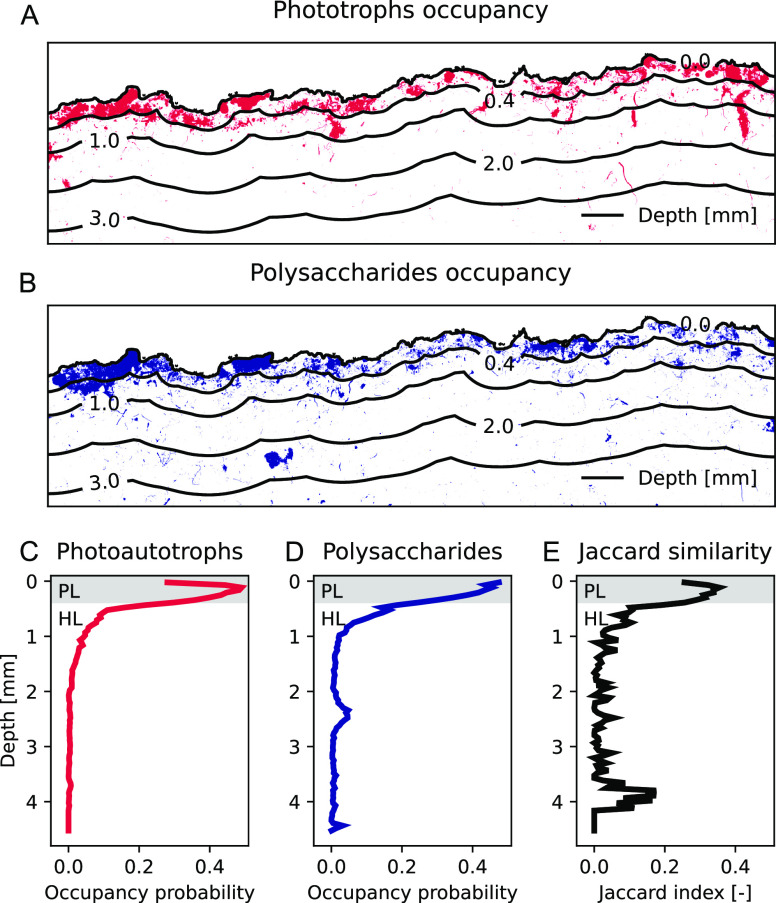
Depth profiles of a cyanobacteria-dominated biocrust cross-section. The red and blue channels of the fluorescence microscopy of the biocrust were used for occupancy maps of (A) photoautotrophic organisms (red) and (B) polysaccharides (blue) based on the minimum cross entropy thresholding. The colored patches indicate the occurrence and extent, the contour lines show the substrate depth. (C,D) The occupancy probability of both parameters were aggregated along soil depth in 50 μm steps. (E) Jaccard similarity index was used to quantify the degree of co-occurrences of photoautotrophs and polysaccharides. The grey shaded area in the profiles indicates the photoautotrophic layer (PL, depth ∼0.4 mm), the area below is considered as the heterotrophic layer (HL, depth >0.4 mm).

Cross-sections revealed a distinctive layer of photoautotrophic organisms in the upper section of the biocrust, but the organisms were not evenly spread but rather concentrated in distinct clumps or hotspots (Figure 2 and S4A,B). In close-up images, a high variability of organism distribution at μm-scale became visible, and polysaccharides were closely associated with photoautotrophic organisms (Figure 2B–E). This was confirmed by occupancy maps of photoautotrophs and polysaccharides obtained by image segmentation with cross entropy thresholding (Figure 3A,B). The occupancy maps illustrate that the photoautotrophic organisms were mainly detected up to a depth of 0.4 mm (thereby defining the photoautotrophic layer, PL) with a notable horizontal variability (Figure 3A,C). A similar pattern was observed for complex polysaccharides, which were mainly concentrated in the uppermost part of the sample (Figure 3B,D). The Jaccard similarity index as a measure of co-occurrence of photoautotrophs and polysaccharides was highest in the PL (Figure 3E).

The heterogeneous distribution of complex polysaccharides, which also form EPS, suggests that they are involved in creating the heterogeneous micro-structure within biocrusts. Previous studies demonstrated that EPS protect microorganisms from ultraviolet radiation (UV) and desiccation, as the EPS matrix may contain UV shielding compounds like Scytonemin^61,62^ and dries slower than its surroundings, thus enhancing their survival in water-deficient environments.^63,64^ EPS are known to maintain hydration by water accumulation and regulation of water loss,^65−67^ thus also contributing to N-cycling processes.^68^ Furthermore, they mediate the adhesion to surfaces, allow an accumulation of nutrients and keep cells in close proximity, facilitating interactions.^63^ In an earlier study it was shown that diazotrophic bacteria can be enriched in the EPS of a non-nitrogen-fixing cyanobacterium, thus influencing its N status.^69^ Our X-ray CT imagery also displayed a strongly heterogeneous pore size structure (Figure 1A,B), which likely causes the heterogeneous colonization of the substrate.

The observed decreasing abundance of photoautotrophic organisms with depth (Figures 2 and 3) is in line with previous reports.^70,71^ The distinctive PL in the upper part of the biocrust (<0.4 mm depth) indicates the penetration depth of light, which leads to the high abundance of chlorophyll_a_. Not only light, but also other factors play a role in the vertical distribution of the photoautotrophs, such as nutrients, water, temperature, and pore structure.^72^ Biocrusts from Utah were colonized by the bundle-forming, filamentous cyanobacterium *Microcoleus vaginatus* Gomont, mainly occurring at 200–500 μm depth, whereas the network of sheaths extended to depths of 3–4 cm.^71^ Also in our study, the polysaccharide concentration decreased somewhat less steeply with depth, but mainly reached to 1 mm depth.

For soils, it has been shown that they generally represent a heterogeneous and dynamic physicochemical environment. They are subject to temporal and spatial variation in the availability of water and nutrients, and the temperature fluctuates in spatially constrained pore spaces. These soil characteristics create microenvironments, differing in water volume, liquid–gas interfacial area, and nutrient availability, resulting in a heterogeneous, patchy distribution of microbes, for instance nitrifiers, in the soil.^73−86^ Similarly, microbial populations are inhomogeneously distributed within biocrusts, mostly as a consequence of vertical gradients in light, oxygen, nutrients as well as water availability, soil properties (texture, pH), and temperature variations.^87,88^ These reports are in accordance with our findings, as we observed a high heterogeneity in the spatial distribution of photoautotrophic organisms in the uppermost soil layer and a high heterogeneity in biogeochemical processes as explained below.

### Biogeochemical Heterogeneity and Fluxes

**Figure 4:**
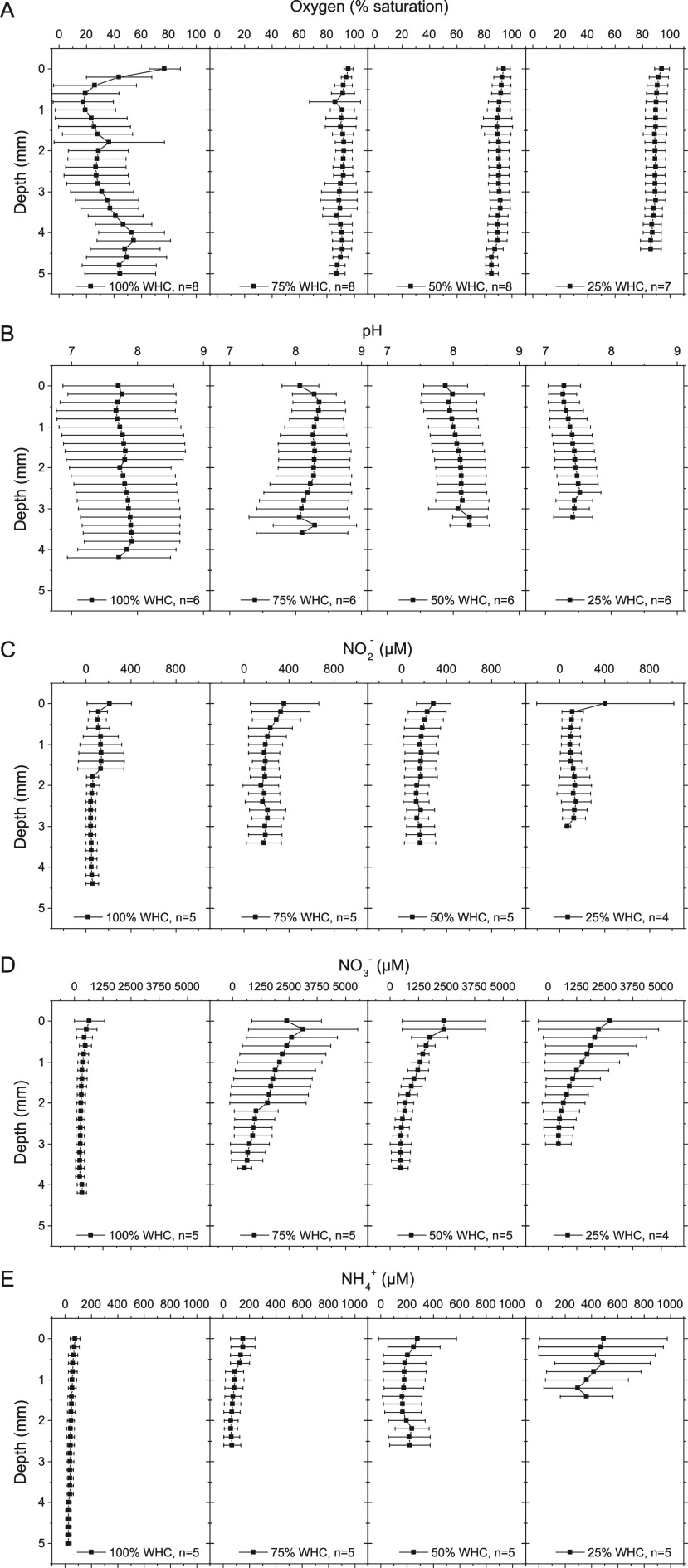
Microsensor profiles of biocrusts assessed at 100, 75, 50 and 25% water holding capacity (WHC) at different locations: (A) oxygen saturation [%]; (B) pH; (C) nitrite (NO_2_^–^) concentration [μM]; (D) nitrate (NO_3_^–^) concentration [μM]; (E) ammonium (NH_4_^+^) concentration [μM]. Error bars indicate standard deviation. For additional information on locations, please refer to Table S3. Locations without changes during desiccation are not included.

**Figure 5:**
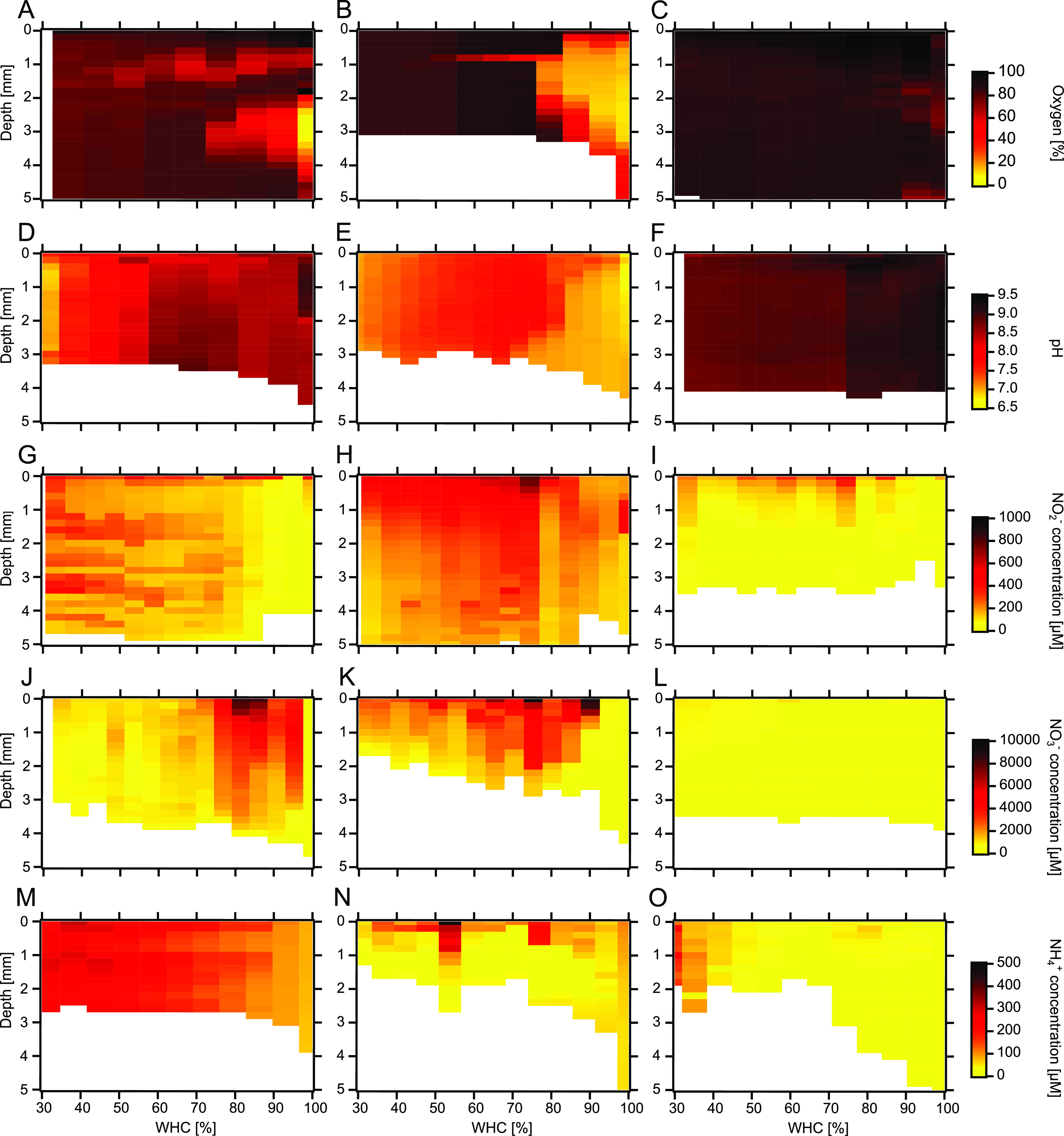
Vertical microsensor profiles (up to a depth of 5 mm) of biocrusts assessed at varying water holding capacity (WHC). (A–C) Oxygen saturation [%], (D–F) pH, (G–I) nitrite (NO_2_^–^) concentration [μM], (J–L), nitrate (NO_3_^–^) concentration [μM], (M–O) ammonium (NH_4_^+^) concentration [μM] in representative biocrust profiles (with the three columns showing measurements at three different locations/puncture sites, reflecting the range of variability) over the course of drying. Profiles were performed at hourly intervals and WHC refers to the whole Petri dish. Additional information on locations measured with different sensor types is given in Table S3. Further measurements are shown in Figure S5.

Oxygen saturation, pH, and N compound concentrations (NO_2_^–^, NO_3_^–^, and NH_4_^+^) were measured using microsensors over the course of desiccation (Figures 4, 5 and S5). In Figure 4, vertical profiles at 100, 75, 50, and 25% WHC are presented, whereas in Figure 5 representative profiles assessed at hourly intervals over the course of a desiccation cycle are shown.

Generally, the microsensor measurements revealed that profiles taken at different locations within biocrust samples differed to a large extent, irrespective of the measured parameter.

**Figure 6:**
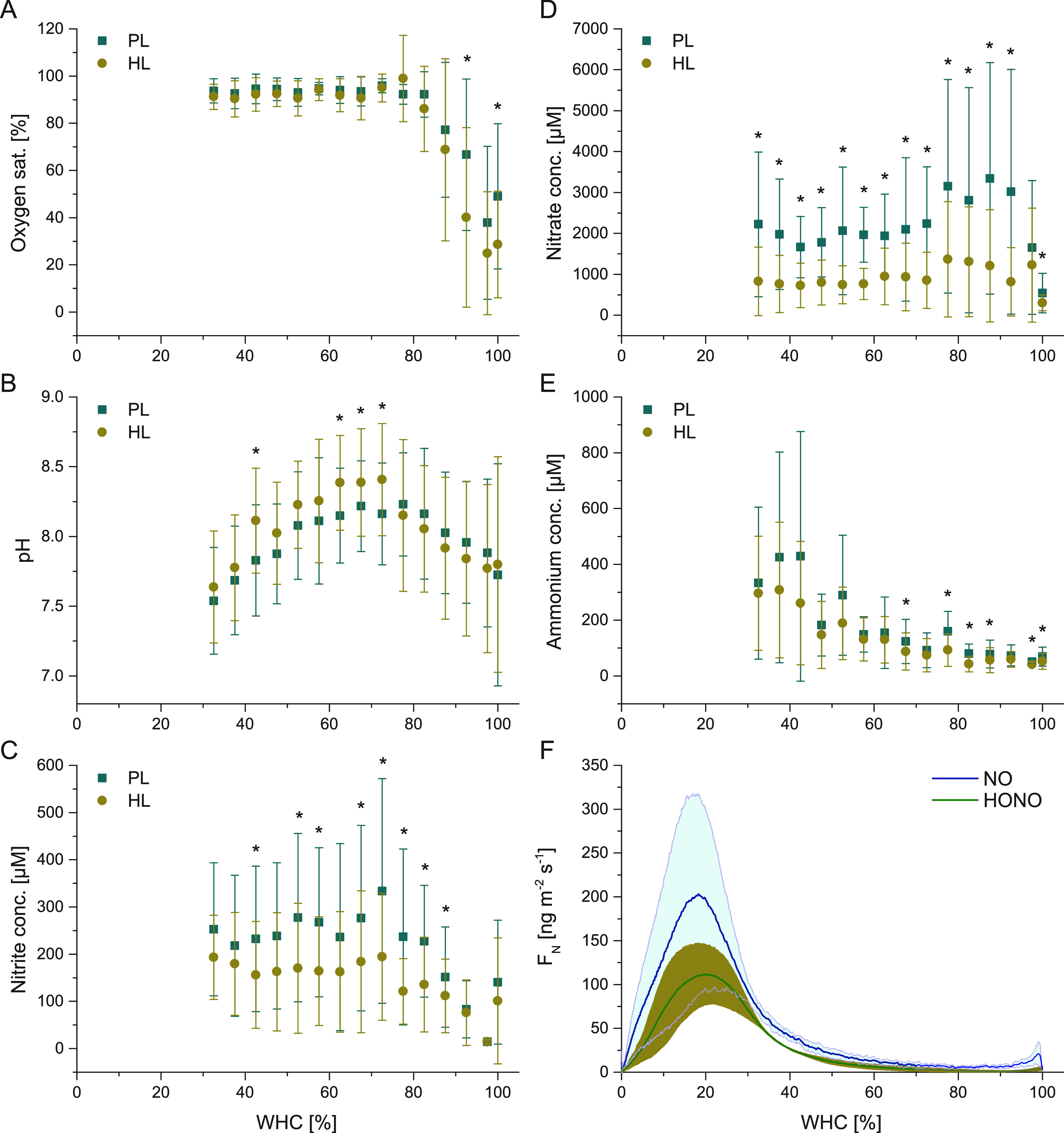
Mean microsensor readings in the photoautotrophic (PL) and heterotrophic layer (HL) and HONO- and NO emissions of biocrust samples as related to the water holding capacity (WHC). (A) Oxygen saturation, (B) pH, (C) nitrite (NO_2_^–^), (D) nitrate (NO_3_^–^), (E) ammonium (NH_4_^+^) content. Measurements were conducted on biocrusts over the course of desiccation and contain several replicates (O_2_: 8; pH: 6; NO_2_^–^/NO_3_^–^/NH_4_^+^: 5). Photoautotrophic layer (PL) covers 0–400 μm depth (200 μm steps obtained by vertical profiles), the heterotrophic layer (HL) starts at 600 μm until the maximum measurement depth; WHC covers the indicated values ± 2.5% (see Methods Section for details). Microsensor profiles without changes during desiccation are not included. Asterisks show significant differences between PL and HL (Statistical results are shown in Table S4) and error bars indicate standard deviation. (F) Average reactive nitrogen emission flux (*F*_N_) of NO and HONO as a function of water holding capacity (WHC). Lines indicate the mean fluxes and shaded areas the standard deviation.

At full WHC, oxygen contents showed high spatial heterogeneity, displaying steeply declining oxygen contents with increasing depth, reaching a mean saturation minimum of 18 ± 22% at 0.8 mm depth. Towards deeper layers the values increased slowly again, reaching a mean saturation maximum of 54 ± 27% at 4.2 mm depth (Figure 4A). Already at 75% WHC, the mean saturation value had increased to an overall value of ∼91 ± 2% with no major differences along depth, indicating an oxygenation of the entire sample that persisted during the subsequent desiccation (Figure 4A and 5A–C). The O_2_-sensors showed lowest O_2_ saturation at high WHC, likely due to the respiration activity of photoautotrophic and heterotrophic microorganisms in combination with the increased diffusion resistance of water as compared to air-filled pores (Figure 4A, 5A–C and 6A). Anoxic conditions in soil occur when the oxygen consumption rate exceeds the oxygen production/transport rate. Especially after hydration, the solubilization of nutrients and the onset of metabolic activity after desiccation stimulate respiration and subsequently result in such anoxic regions.^89^ Our results suggest that the microbial respiration was sufficient to create anoxic regions, even in the upper region close to the interface with the atmosphere. This was similarly observed in earlier studies, where anoxic areas occurred from the surface to several millimeters depth in fully saturated biocrusts.^71^ Such events of full water saturation with anoxic conditions do not occur frequently in drylands, but mesoclimate data assessed during the BIOTA project (www.biota-africa.org) and during measurements of ourselves (B. Weber, unpublished) show that daily precipitation reaching 10–20 mm normally occurs on several occasions per year. During these occasions, we expect at least nearly full water saturation to be reached over short time-spans. As soil dries, gaseous diffusion is facilitated and allows oxygenation of the soil.^76^ We observed that both the photoautotrophic and heterotrophic layers became fully oxygenated when the biocrust sample dried to ∼70% WHC (equivalent to a water content of 0.09 g * g^–1^; Figure 6). However, in this context it has to be considered that, because of the measurement conditions (darkness), no photosynthesis occurred. In former studies, photosynthesis in illuminated biocrusts recovered within minutes after hydration, resulting in the formation of an oxygen-supersaturated zone close to the surface and anoxic zones at 1–3 mm depth.^90^

The microsensor measurements also revealed not only vertical but also horizontal microscale heterogeneity. Spatially restricted areas and limited periods of oxygen-limited conditions over the course of desiccation (Figure 4A and 5A–C) are likely caused by the patchy distribution of microbial cells and EPS (Figure 2) causing variations in oxygen production/consumption and transport. Previous studies of desert biocrusts have also shown that respiration in cyanobacterial crusts started within minutes upon hydration, and, in line with our observations, it was concluded that disparate chemical microsites had formed,^71,90,91^ which were also suitable for anaerobic processes, such as anaerobic methanogenesis in crusts from the Negev Desert^92^ and denitrification in crusts from Oman.^10^

Average pH values increased from 100 to 75% WHC with a mean maximum of 8.4 ± 0.4 at 0.4 mm depth. During subsequent desiccation, the mean values decreased again, maximum values shifted towards deeper layers (∼2–3 mm depth), and at 25% WHC the near-surface pH (7.2 ± 0.3) was lower than the initial value at full WHC (7.7. ± 0.8; Figure 4B). During individual microsensor measurements, the pH values ranged between ∼6.5 and ∼9 at different locations and desiccation stages (Figure 5D–F and S5). Whereas in some locations pH values decreased considerably over the course of desiccation (from ∼9.0 to ∼6.9; Figure 5D), in other spots nearly no changes in pH were observed over the course of desiccation (Figure 5F). We detected locations with high pH values at the beginning of the measurement that decreased over the course of desiccation, which may be caused by chemical reactions and biological processes. In former measurements, variable pH values were reported for bulk samples of different dark cyanobacteria-dominated biocrusts from the Succulent Karoo and Cyprus, ranging from 6.8 to 8.0 and 6.8 to 7.3, respectively.^18,20^

Mean NO_2_^–^ concentrations revealed an increase and a subsequent decrease from 86 μM at 100% WHC to 212, 174, and 133 μM at 75, 50, and 25% WHC, respectively (Figure 4C). At 100% WHC, highest mean NO_2_^–^ concentrations of 136 μM were reached at 1–1.6 mm depth, whereas subsequently the highest mean values occurred at the surface. Lowest standard deviations were observed at 100% WHC at 1.8 mm depth and below, whereas the highest value occurred at the surface at 25% WHC. Individual measurements showed large variation, as some of them were characterized by increasing concentrations at specific depths (Figure 5G), others showed increased concentrations towards the biocrust surface, with a stable pattern throughout the desiccation process (Figure 5H), and a third group displayed no major changes in deeper layers and only somewhat higher concentrations close to the surface (Figure 5I). The initial increase in NO_2_^–^ was probably due to the onset of spatially localized microbial activity upon hydration. Recent work has shown that soil bacteria respond within hours to days to an increase in soil water availability after prolonged drought.^93−95^ The quick microbial response is associated with CO_2_ emissions^93^ and an increase in transcript copies of bacterial *rpoB* genes, encoding bacterial RNA polymerase, indicating resumption of transcriptional activity.^96^

Average NO_3_^–^ concentrations were lowest at full WHC (337 ± 108 μM) and highest at 75% WHC (1572 ± 766 μM) (Figure 4D). With progressing desiccation, there was a slight reduction in maximum values, and generally mean concentrations were high close to the surface and showed a strong decrease towards deeper strata. Such high surface values were also observed in individual measurements, but only in one representative sample, this stratification lasted until desiccation (Figure 5K), whereas in another sample a stratification dissolved below 70% WHC (Figure 5J). Also for NO_3_^–^ there were measurements where no major changes were observed along desiccation (Figure 5L).

Ammonium concentrations were lowest at full WHC and increased over the course of desiccation, accompanied by increasing standard deviations (Figure 4E). Generally, the highest concentrations occurred close to the surface, but at 50% WHC slightly increased concentrations were also observed at ∼2.4 mm depth. In some individual sensor measurements, the increasing ammonium concentrations along desiccation could be nicely observed (Figure 5M), whereas in others increased values occurred mainly towards the surface, but were otherwise fairly stable (Figure 5N). In other measurements, increased values were only observed towards the end of desiccation (Figure 5O).

Generally, soil water content has been identified as the main variable that controls the pore space, hence shapes diffusion of oxygen and other nutrients.^97^ The small-scale structural heterogeneity of the substrate and its pore space, which has been observed during X-ray microtomography, thus explains the variability of oxygen and nutrient contents at the micro-scale. This availability of oxygen and nutrients to microorganisms has been shown to influence the rates and patterns of biogeochemical processes, which could cause emissions of CO_2_, N_2_O, and CH_4_ from soil aggregates.^80,98−100^ During desiccation, we observed that only the concentration of ammonium showed an increase, whereas that of NO_2_^–^ and NO_3_^–^ remained fairly stable, indicating a conversion of both nitrogen species. An explanation for the NO_2_^–^ loss could be its emission as HONO or NO from the biocrust.^20^ The loss of NO_3_^–^ could be due to denitrification.

### Microsensor Data Related to Dynamic Chamber Measurements

Although the microsensor technique allowed measurements only until 30% WHC (but not below), these data still could be related to online chamber flux measurements that showed increasing N_r_ emissions at 30% WHC (Figure 6). Oxygen saturation values of fully wetted samples increased strongly until 80% WHC, with higher values in the PL as compared to the HL, and stayed similarly high until desiccation (Figure 6A). This was different for the pH, which first increased until a WHC of ∼70% and then showed a strong decrease again, reaching a mean pH of ∼7.6 (Figure 6B). This final decrease of pH coincided with increasing HONO and NO emissions (Figure 6F). This is in line with the mechanistic soil model predictions of Kim and Or,^100^ who suggested that changes in aqueous film thickness and a local decrease in pH during desiccation drives the emission of HONO.^100,101^ The model prediction also supports our findings by demonstrating that mean water content and bulk soil pH values may not capture the nuances at microscale associated with efflux patterns, like the HONO emissions from alkaline soils or the concurrent emissions of NH_3_ and HONO during desiccation cycles.^100^

Also the mean NO_2_^–^ concentrations increased until ∼70% WHC (PL up to 350 μM and HL up to 200 μM), which was followed by a slight decrease in the PL and constant values in the HL (Figure 6C). Mean NO_3_^–^ concentrations were highest at high water contents of ∼80–90% WHC (PL up to 3500 μM and HL up to 1500 μM) and subsequently they stabilized at values around 2000 μM in the PL and around 750 μM in the HL (Figure 6D). For both NO_2_^–^ and NO_3_^–^ the values were mostly significantly higher in the PL as compared to the HL (Figure 6C,D). Mean ammonium concentrations increased with progressing desiccation accompanied by increasing standard deviations (Figure 6E). Mean HONO and NO values showed a maximum flux of 112.57 ± 113.32 ng m^–2^ s^–1^ HONO–N and 205.34 ± 34.27 ng m^–2^ s^–1^ NO–N, respectively, at ∼20% WHC (Figure 6F). Thus, the concentrations of N-compounds were also highly variable in space and time, but generally the lowest values occurred at full WHC and increased towards 75% WHC with subsequently decreasing NO_2_^–^, increasing ammonium, and stable NO_3_^–^ values (Figure 6C–E). This fits to the observation that lower pH values are associated with decreased rates of ammonia oxidation (AO), leading to a decrease in NO_2_^–^ at lower WHC.^102^ Interestingly, concentrations of NO_3_^–^ were generally higher than those of NO_2_^–^, which is in line with NO_2_^–^ and NO_3_^–^ contents obtained for complete biocrust samples.^32^ The highly variable measurement results indicate that biotic processes, such as nitrification and denitrification, with NO_2_^–^ as an intermediate product and precursor of HONO, are spatially restricted. Microsites with high reaction rates compared to the surrounding area have been observed in soil, where a nonhomogeneous distribution of denitrification activity has been identified.^103,104^ In future studies, it would be desirable to investigate the same samples with different techniques, for example X-ray CT and fluorescence microscopy, in order to have a direct link between structural and organismic composition, or fluorescence microscopy and microsensor measurements, in order to link organismic and nutrient composition.

Overall, our study revealed that the biocrust substrate structure consisted of a stable mix of pore size classes, the distribution of organisms was patchy, and the ion concentrations occurred in strongly heterogeneous patterns. Our results suggest that the highly variable biocrust structure allows the formation of spatially separated microhabitats, where different, highly dynamic and even contradictory soil N transformations occur simultaneously within millimeter distances during drying. This knowledge needs to be considered in future global change and land management scenarios.

## Supporting Information Available

Methods (Sampling, X-ray microtomography, Fluorescence microscopy, LIX sensors, Dynamic chamber measurements), Water holding capacity (%) of a biocrust (Figure S1), X-ray microtomography images of a biocrust sample (Figure S2), Porosity profiles of a biocrust sample (Figure S3), Fluorescence images showing photoautotrophic organisms and polysaccharides (Figure S4), Microsensor profiles of oxygen saturation, pH, and N compound concentrations (Figure S5), Information on X-ray micro-CT (Table S1-S2), Information on microsensor measurements (puncture sites, date of measurement, Table S3), Results of statistical analysis for microsensor measurements (Table S4) (PDF)
