# Supplementary material for: Reactive Nitrogen Hotspots Related to Microscale Heterogeneity in Biological Soil Crusts
Source: Environ Sci Technol. 2022 Aug 5;56(16):11865–77. doi: 10.1021/acs.est.2c02207 (PMC9387110; doi:10.1021/acs.est.2c02207)
Supplement: Supplementary file 1 — es2c02207_si_001.pdf [file es2c02207_si_001.pdf]

Supplementary Material for manuscript

# Reactive nitrogen hotspots related to microscale heterogeneity in biological soil crusts


Alexandra Maria Kratz*[+1], Stefanie Maier[+1,2], Jens Weber[+1,2], Minsu Kim[2], Giacomo Mele[3], Laura Gargiulo[3], Anna Lena Leifke[1], Maria Prass[1], Raeid M. M. Abed[4], Yafang Cheng[1], Hang Su[1], Ulrich Pöschl[1], Bettina Weber*[1,2]

1 Max Planck Institute for Chemistry, Multiphase Chemistry Department, 55128 Mainz, Germany.

2 Institute of Biology, Division of Plant Sciences, University of Graz, 8010 Graz, Austria.

3 Institute for Agriculture and Forestry in the Mediterranean, National Council of Research, 80055 Portici, Italy.

4 Sultan Qaboos University, College of Science, Biology Department, PO Box 36, Al Khoud 123, Sultanate of Oman.

[+]These authors contributed equally to this work.

*Corresponding authors


Contents


Methods .......... 1

Sampling .......... 1

X-ray microtomography .......... 1

Fluorescence microscopy .......... 2

Production and calibration of liquid ion-selective membrane sensors (LIX) .......... 3

Microsensor measurements .......... 4

Calibration of sensors .......... 5

Dynamic chamber measurements .......... 5

Figures (S1-S5) .......... 6

Tables (S1-S4) .......... 11

References .......... 13

# Methods

## Sampling

Biocrusts generally are classified into cyanobacteria-, lichen-, and moss-dominated types, according to the dominating photoautotrophic organism. Cyanobacteria-dominated biocrusts at our sampling site mostly grew together with lichens of the species *Collema coccophorum* Tuck. and comprised the cyanobacterial genera *Microcoleus, Pseudoanabaena, Chroococcidiopsis, Phormidium, Leptolyngbya,* and *Nostoc*. Lichen taxa were classified based on morphological features, the dominating cyanobacteria have been identified in former studies by means of both morphological and genetic identification techniques [1]. 40 samples of cyanobacteria-dominated biocrusts were collected in the field, whereof one sample was used for X-ray CT, one for fluorescence microscopy (3 different cross-sections) and 20 for microsensor measurements. Details on the sensor composition during microsensor measurements are given in table S3. We could not use all the samples, since some of them were destroyed during transport.

## X-ray microtomography

The dry weight was determined by drying the sample in an oven at 41°C for 24h. Higher temperatures were avoided to preserve the overall structure and organic matter in the biocrust. In table S2, the weights and the corresponding gravimetric water contents and saturation degrees are reported. In order to obtain a good x-ray scan without imaging artefacts, ~50% WHC (~80% of the field capacity (FC)) could not be exceeded. Indeed, the state of saturation was quite unstable and only at ~50% WHC, the desiccation rate was sufficiently slow to relate a given hydration status to the reconstructed soil structure without small crust movements during scanning time.

Reconstructed biocrust volumes at the two distinctive hydration states were analysed by applying the "successive opening" 3D image analysis procedure [2] to the pore phase. This allows the segmentation of the pore volume according to the pore wall spacing, using the Conmorph software [3].

To explore the structural heterogeneity within the investigated sample, the reconstructed image of the whole biocrust volume at dry state was subsampled at nine different locations. Nine thin columns, each of ~0.64 $mm^2$ surface area and ~4.5 mm height, were virtually extracted from the biocrust sample and vertical porosity profiles were determined. The 8-bit grey level reconstructed images obtained from micro CT scans were segmented by applying a single global level automatic threshold. The grey level threshold was identified by the Otsu method[4] implemented in the CT Analyzer software v. 1.19 (www.bruker.com). It finds thresholds that minimize inter-class variance of the grayscale histogram. The procedure provided 50 and 35 as grey level thresholds for dry and wet state images, respectively.

## Fluorescence microscopy

For microscopy, the samples were embedded in 0.9 % agarose and incubated for 10 h in the dark to allow the recovery of chlorophyll. Subsequently, the samples were fixed in 4% paraformaldehyde (PFA) in phosphate-buffered saline (PBS) solution for 60 min and then washed with 1x PBS to remove excess PFA. Prior to cutting, overview images of the samples were taken with a binocular (Zeiss, Oberkochen, Germany) and the installed camera (Axiocam color, Zeiss, Oberkochen, Germany). Samples were cut from the bottom to the top of the biocrust to avoid a mechanical spreading of the photoautotrophic organisms to deeper biocrust strata.

Stained sections were mounted on glass slides with glycerol and covered with coverslips. The samples were analyzed using a Nikon Ti2-E inverse research microscope (Nikon, Tokyo, Japan) equipped with an LED light source (Lumencor Sola SE II 365HBO, Nikon) and an APO 10 x object lens (Nikon). For image recording, a camera (DS-Qi2, Nikon) operated with an imaging software (NIS-Elements, Nikon) was used.

Signals of chlorophyll autofluorescence were detected with a MXR00710 TxRed-4040C filter cube (Ex/Em 562/624 nm). UV excitation and a MXR00700 DAPI-1160B filter cube (Ex/Em 387/447 nm) were used to visualize polysaccharides stained with Calcofluor-White (CFW). CFW binds to 1,3 beta and 1,4 beta bonds in polysaccharides. These bonds are e.g. present in chitin and cellulose [5]. In the sections, CFW also stained the area above the section, which was covered by glycerol used to mount the samples on the slides for microscopy. This background noise is likely caused by i) refraction of the light emitted by CFW stained EPS in the glycerol layer, and ii) slow dissolution of small extractable EPS parts in the glycerol, as the color intensity of the background increased with time after mounting. For detection of soil particles, a F46-018NXL EGEP ET lowpass filter set (Ex/Em 470/500 nm) was used.

For image generation, the stacking and stitching options of the software were used. The microscope generated a staple of ~100 levels, and at each level, an overall area of 22 x 8 mm was scanned. At each level, images next to each other were combined by stitching. The images taken at each level were composed of three false-colour images taken with the three different filter sets. All levels were combined using the EDF (extended depth of focus) function to generate one image with maximum sharpness and exported as TIFF files.

The TIFF files were colour corrected and saved as jpeg using Irfanview (Version 4.57). Images were binarized using ImageJ (Version 1.52e); black was set as background and white as signal. From the three overview images, 20 sections of 1 mm width, covering the complete height of the image, were extracted for further analyses. For an analysis of the distribution of photoautotrophs and complex polysaccharides, the red and blue channels were used. Occupancy maps were generated by using Li thresholding (implemented in skimage.filters.thredhold_li, Version 0.20), which minimizes the cross-entropy between the foreground and the foreground mean, and the background and the background

mean [6-7]. Euclidian distance from the surface was used as the depth and the values were calculated at 50 µm intervals.

## Production and calibration of liquid ion-selective membrane sensors (LIX)

Methods and the production procedure are described by de Beer, et al. [8,] de Beer and Sweerts [9,] de Beer, et al. [10,] de Beer and van den Heuvel [11], and Revsbech and Jørgensen [12] and below. The LIX microsensors are not commercially available due to their short lifetime and cannot be used in brackish or marine waters due to interferences (high salt concentrations). Green soda lime glass capillaries (3.5 mm outer diameter, No. 8516; Schott, Mainz, Germany) with good insulating characteristics were heated in the flame of a Bunsen burner and pulled into thinner capillaries. The shoulder at the thick part of the green glass was cut using a glass cutter and then the thin part was fused to a slightly tapered white glass (5 mm outer diameter, AR glass, Schott, Mainz, Germany) in a small flame. Both glass parts had to be completely sealed. In order to confer more stability, the sensor tips were thickened and cut with a diamond knife at the requested diameter of about 20-30 µm (inner diameter) under the microscope. The glass surface of the capillaries was silanized to obtain a hydrophobic surface for optimal adherence of the LIX membrane. The capillaries were put into a desiccator and baked in an oven (at least 3 h at 150°C to remove traces of water). Then, 200 µl silanizing agent (N-dimethyltrimethylsilylamine) were added to the desiccator, the opening of the desiccator was quickly closed with the help of a plug and left overnight at a temperature of 200°C. On the following day, the plug was removed to release the excess silane vapor (under fume hood) and the capillaries were baked for another 2 h at 150°C to clean off the remaining silane. The LIX membrane was filled into the silanized tip of the capillary via the tip of a tapered Pasteur pipette. For this, the two tips were placed face to face of each other and under a microscope, with the help of a micromanipulator, where the Pasteur pipette was fixed, the position was carefully adjusted. Using a syringe connected to a tubing, which was pulled over the backside of the capillaries, the LIX membrane was poured into the capillary tip by applying suction with the syringe until a width of the LIX membrane obtained at least 300 µm. In a first step the LIX membrane without and in a second step the membrane with PVC was added. The PVC containing membrane improves the stability and performance of the LIX microsensors due to its viscous properties. After at least one night of drying, just before the measurements, the corresponding electrolyte, which finally activates the sensors, was filled into the capillaries. All air bubbles on the surface of the LIX membrane had to be removed to guarantee an electric circuit. In order to reduce electrical noise of the sensor, the silanized glass capillaries were inserted into an outer glass casing, which was filled with an electrolyte (3 M KCl). The 3M KCl solution (casing/coaxial shielding) was then connected to the reference using a chlorinated silver (Ag/AgCl) wire, which is much cheaper and even more efficient than a Faraday cage [13]. In a last step, a thinner silver wire (Ag/AgCl; 50–200 µm in diameter) was soldered to a coaxial cable to connect the

electrolyte and the multimeter (Version2.01, Unisense, Denmark), thus enabling the detection of the signals (mV). Noise sensitivity of the sensors was effectively avoided by shielding, which functioned much better and was more convenient than a Faraday cage [8]. Good silanization guaranteed attachment of the liquid membrane to the inner glass walls of the sensor tips and could counter ion shunts to some extent [8, 14]. An increase of the tip diameter improved the mechanical strength of the microsensor and also countered ion shunts, but since the capillary force was reduced, the membrane had to be stabilized in the tip by gelation with PVC [8]. In order to confer more stability for the ion-exchange membranes in the sensor tip, an important adaption was the thickening of the outer glass walls. For this, the tips of the microcapillaries were thickened, whilst they were initially put into the flame of the Bunsen burner, then they were horizontally compressed and finally slightly stretched. After that, they were cut with a diamond knife at the requested diameter of about 20-30 µm (inner diameter) under the microscope. With exception of the pH microsensors, limited selectivity of ionophores required calibration in an experimental medium [8].

## Microsensor measurements

Measurement series were conducted in 2017, 2018 and 2019. In all measurement series, LIX-sensors with a tip diameter of 20-30 µm were used for the determination of $NO_3^-$, $NO_2^-$, and $NH_4^+$ concentrations. For pH measurements, LIX sensors were used in 2017, whereas in 2019 pH microelectrodes were applied (pH-100, Unisense A/S, Aarhus, Denmark). The pH microelectrodes (solid-state electrodes) were based on ion-selective glasses, whereas the pH LIX microsensors (liquid-membrane electrodes) were based on organic ion-exchangers [12]. $O_2$ saturation was analyzed with the help of oxygen microsensors. Oxygen microsensors are based on diffusion of oxygen through a silicone membrane to an oxygen reducing cathode, polarized against an internal Ag/AgCl anode (OX-100, Unisense A/S, Aarhus, Denmark).

Since the microsensors showed a drift during the measurement period, they had to be calibrated before and after each measurement cycle using standard solutions. Besides that, the height had to be adjusted before each profile (Supplement, table S4). The profiles were measured at discrete depth intervals of 200 µm to a maximum depth of 5 to 6 millimeters. The different locations as well as consecutive vertical profiles differed in the maximum depth. This was caused by a variation of the biocrust surface height. Over the course of the measurements, the procedure was slightly adapted based on the experiences made during the first measurements.

During the first two measurement series (2017, 2018), the sensors remained in a safe position above the biocrust surface in air between two profiles, whereas in later measurements (2019) they were placed at a depth of 3000 µm in between. We experienced that sensor lifetime could be extended by placing them within the biocrust between measurements. During the first measurements, weight was consistently logged during desiccation, using a weight balance placed underneath the samples.

However, it was observed that sensor movement distorted the balance readings. Thus, subsequently the weight was interpolated linearly between the start and end weight, which proved to be sufficiently accurate to reflect the water content during a desiccation cycle.

The photoautotrophic layer (PL) covered 0-400 µm depth (200 µm steps obtained by vertical profiles), the heterotrophic layer (HL) started at 600 µm and ranged until the maximum measurement depth (3400 µm, 4400 µm, 2400 µm, 4000 µm, 2000 µm, for pH, oxygen saturation, nitrite, nitrate, and ammonium, respectively; Fig. 4).

## Calibration of sensors

Absolute concentrations were calculated based on a standard curve. The pH LIX, as well as the pH microelectrodes (solid-state electrodes) were calibrated utilizing a two-point calibration with buffer solutions at pH 7 and 9 (pH 7 Fixanal; pH 9 buffer solution (20 °C) colored; Honeywell Fluka, Seelze, Germany). Nitrate, nitrite, and ammonium microsensors were calibrated using standard concentration series (5 µM, 10 µM, 50 µM, 100 µM, 500 µM, 1mM; in some cases additional steps, e.g. 10 mM and 100mM were added) obtained from stock solutions ($NH_4^+$ - stock solution: 330,35 mg $(NH_4)_2SO_4$/ 25 ml = 100mM; $NO_2^-$ - stock solution: 172,5 mg $NaNO_2$ / 25 mml = 100 mM; $NO_3^-$ - stock solution: 252,775 mg $KNO_3$/ 25 ml = 100 mM). Oxygen microsensors were calibrated using a two-point linear calibration with air saturated water and $O_2$ depleted water gained by vigorous bubbling with oxygen free inert gas ($N_2$; Oxygen sensor user manual; Unisense A/S, Aarhus, Denmark).

## Dynamic chamber measurements

HONO was detected spectrophotometrically, using a long path absorption photometer (LOPAP, QUMA Elektronik & Analytik, Wuppertal, Germany; total accuracy 10% and detection limit ~3-6 ppt), whereas NO and $NO_2$ were analyzed with a gas chemiluminescence detector (equipped with a blue light converter; Model 42C, Thermo Electron Corporation, USA; limit of detection $(LOD)_{NO} \approx$ 120 ppt and $LOD_{NO2} \approx$ 300 ppt) [15-16]. An acidic solution of sulphanilamide is used to sample HONO with a stripping coil directly connected to the chamber [15-19]. Upon reaction, HONO is immediately transformed into a diazonium salt, which serves as a precursor of a diazotization causing the formation of an azo dye. The concentration of the azo dye, which is equivalent to the concentration of HONO in the sampled air, is determined by means of VIS-spectrophotometry. A detailed description of the instrument was presented by Heland, et al. [20] and Kleffmann, et al. [21]. In simplified terms, NO was detected with the help of the chemiluminescence reaction ($NO + O_3 = NO_2 + O_2 + h\nu$), whereby a characteristic luminescence is produced, which can be measured by an integrated Photomultiplier tube (PMT;Model42i).

# Figures (S1-S5)

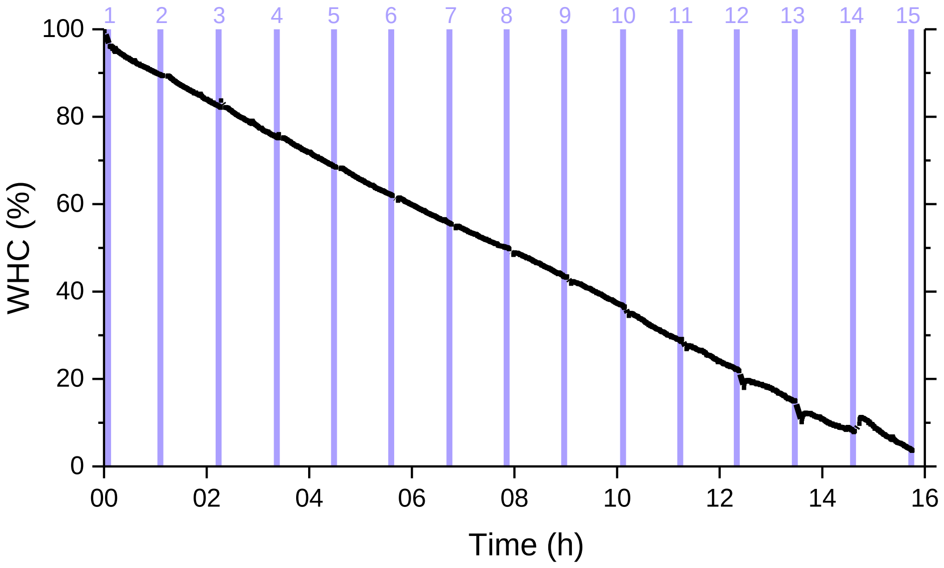


**Fig S1:** Representative curve of water holding capacity (WHC; %) of biocrust along desiccation cycle analyzed over time (h). The purple shaded areas illustrate the times when profiles were measured.

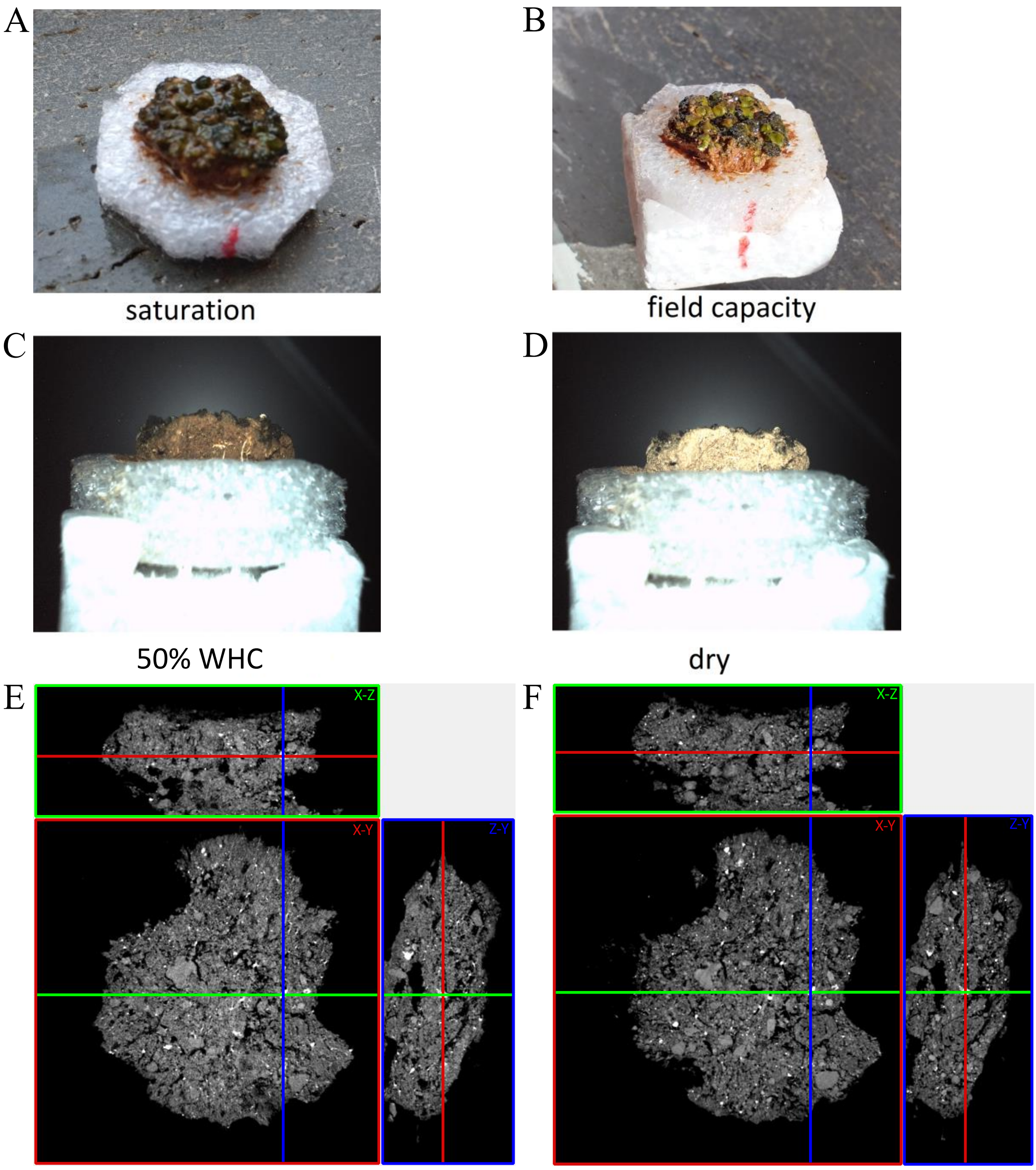


**Fig. S2:** Biocrust sample at different hydration states. A: oversaturated; B: saturated until field capacity (FC); C: 50% water holding capacity (WHC; corresponding to 80% FC); D: dry condition; E, F: X ray micro CT images of orthogonal sections of the crust sample at (E) 50% WHC and (F) under dry conditions. The horizontal cross-section is marked and the resulting image framed in red, the two vertical cross-sections and resulting images are marked and framed in green and blue, respectively.

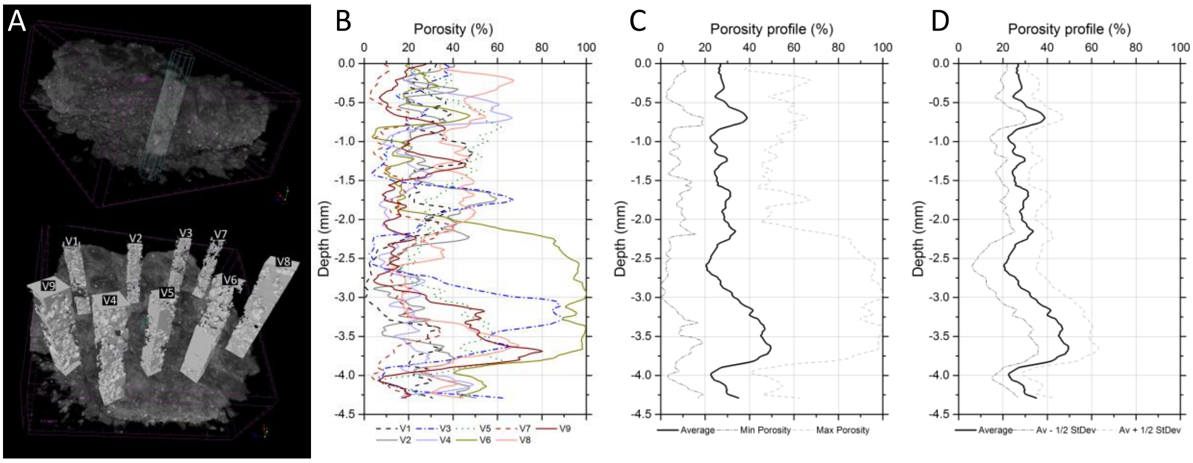


**Fig. S3:** Biocrust subsampling in regions of interest (ROIs): (A) Illustration of the nine ROIs. Pores are shown in white color in the binarized ROIs named V$i$ ($1 \leq i \leq 9$); (B) Vertical porosity profiles of 9 ROIs; (C) Average, minimum (Min), and maximum (Max) porosity profiles; (D) Average, average - ½ standard deviation (StDev), and average + ½ standard deviation (StDev) porosity profiles.

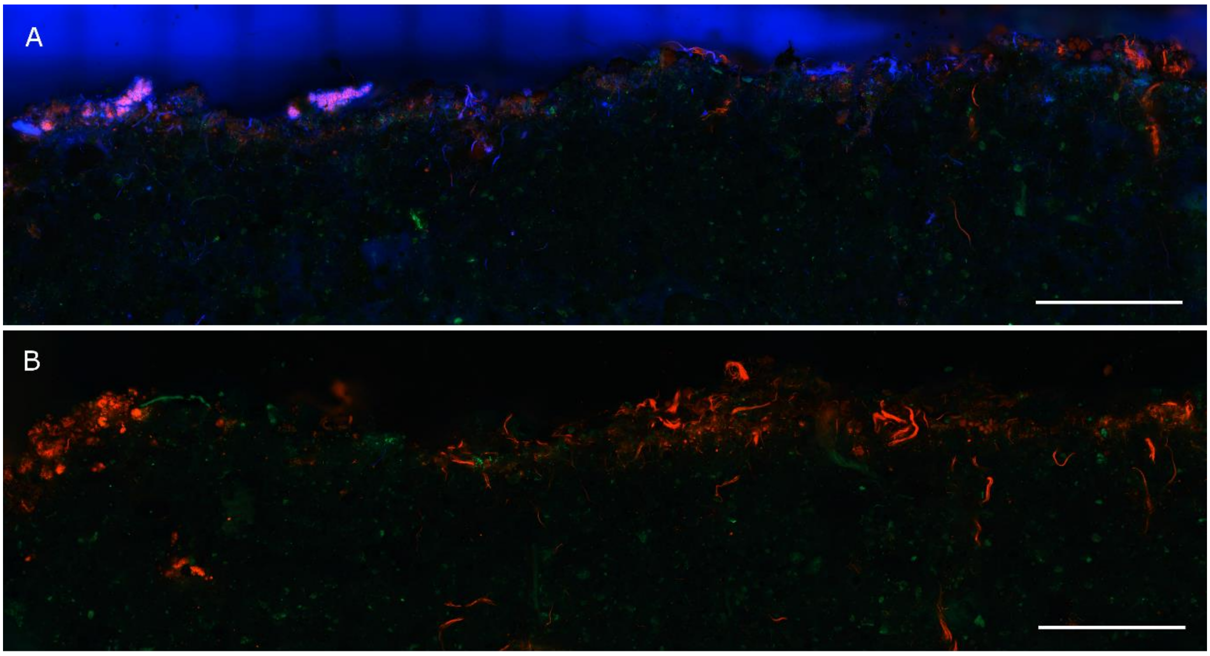


**Fig. S4:** Merged fluorescence micrographs of cross-sections of cyanobacteria-dominated biocrusts. The red channel shows photoautotrophic organisms; polysaccharides (like chitin, cellulose and cyanobacterial extracellular polymeric substances (EPS) are shown in blue, and pedological features (e.g., sand grains, stones) in green color. In (A) all three channels are shown, whereas in (B) only the red and green channels are presented. Scales represent 2.5 mm. Additional illustrations are shown in Fig. 2.

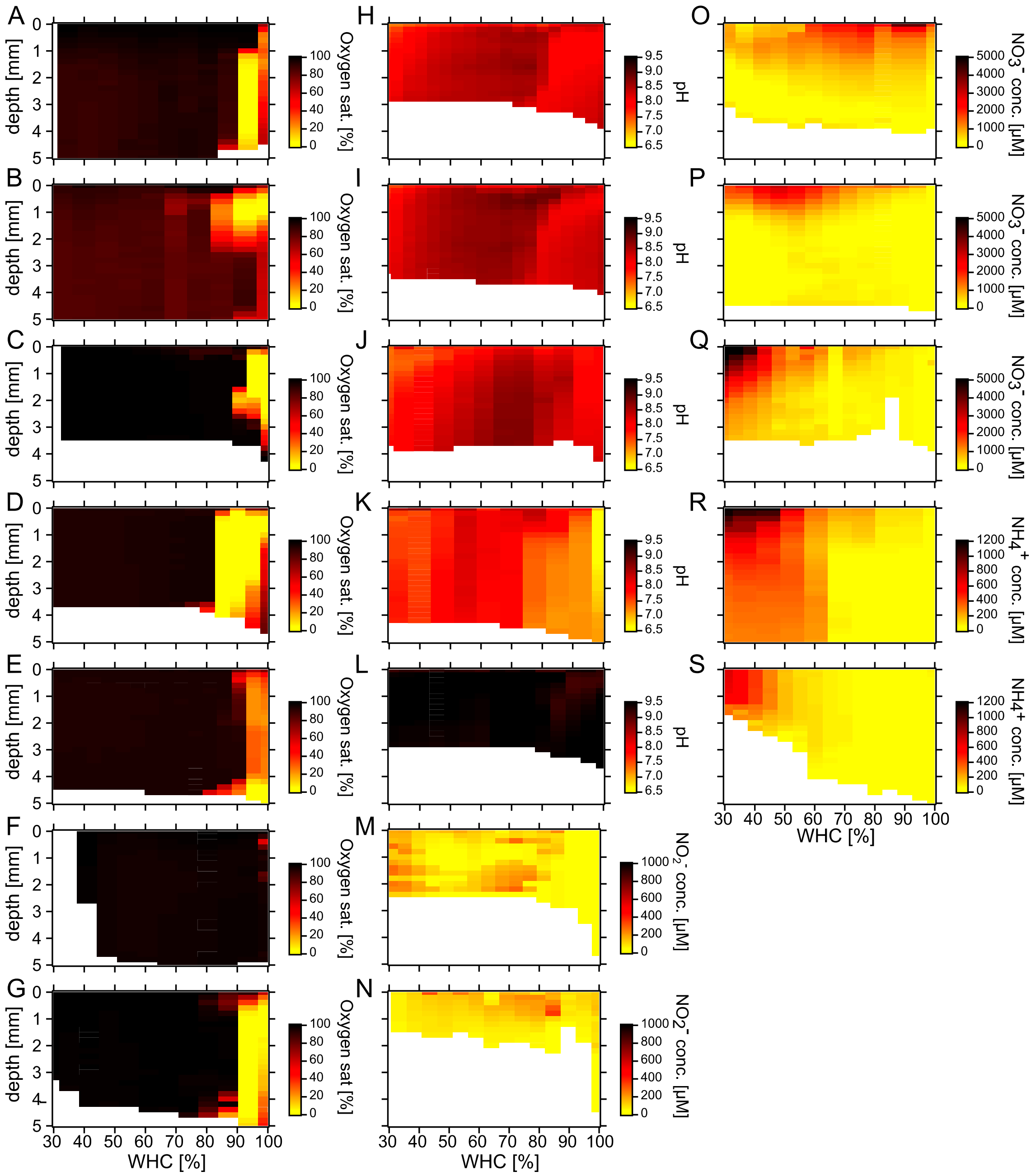


**Fig. S5:** Individual vertical microsensor profiles. (A-G) Oxygen saturation [%], (H-L) pH, (M-N) nitrite concentration [µM], (O-Q), nitrate concentration [µM], (R-S) ammonium concentration [µM]. Additional profiles are shown in Fig. 5. Table S3 shows information on individual locations.

# Tables (S1-S4)

**Table S1**: Scanning protocol and reconstruction for x-ray micro-CT.

| A: Scanning protocol | | B: Reconstruction | |
|---|---|---|---|
| Source Voltage (kV) | 100 | Smoothing level | 10/30 |
| Filter | Cu 0.11mm | Smoothing kernel | 2 |
| Exposure (ms) | 2871 | (Gaussian) Ring Artifact Correction | 20/50 |
| Rotation Step (deg) | 0.200 | Beam Hardening Correction (%) | 70 |
| Frame Averaging | ON (6) | Filter type description | Hamming (α = 0.54) |
| Image pixel size (µm) | 10 | Cone-beam Angle Horiz. (deg) | 9.355403 |
| | | Cone-beam Angle Vert. (deg) | 6.264974 |
| | | Voxel size (µm) | 10 |

**Table S2**: Information of subsample for X-ray microtomography: Weight, soil water content, and water holding capacity (WHC) under different hydration conditions.

| | Weights (g) | Net weights (g) | Soil water content (gravimetric, %) | Water holding capacity (WHC; gravimetric, %) |
|---|---|---|---|---|
| tare | 0.14 | 0,00 | | |
| Sample state | | | | |
| initial | 2.32 | 2.18 | 0.32% | 1.59% |
| at saturation | 2.75 | 2.61 | 19.97% | 100.00% |
| at Field Capacity (FC) | 2.60 | 2.45 | 12.95% | 64.85% |
| 80% FC | 2.53 | 2.39 | 9.95% | 49.82% |
| Dry (oven-dry) | 2.32 | 2.17 | 0.00% | 0.00% |

**Table S3**: Information on locations/puncture sites measured with different sensor types, the date of measurement (Date: year/month/day), and the representative figure number (Fig.). In most cases different sensor types were placed in one sample but different locations/puncture sites.

| Loc. | Oxygen | | pH | | $NO_2^-$ | | $NO_3^-$ | | $NH_4^+$ | |
|---|---|---|---|---|---|---|---|---|---|---|
| | Date | Fig. | Date | Fig. | Date | Fig. | Date | Fig. | Date | Fig. |
| 1 | 20190221 | S5C | 20190221 | 5F | 20181217 | S5M | 20190221 | 5J | 20190301 | 5O |
| 2 | 20190224 | S5D | 20190224 | S5L | 20181218_1 | 5I | 20190224 | 5K | 20190306 | 5N |
| 3 | 20190225 | S5E | 20190225 | 5E | 20181218_2 | 5G | 20190225 | S5Q | 20190307 | S5S |
| 4 | 20190227 | S5F | 20181119_1 | S5H | 20181218_3 | S5N | 20181210_1 | 5L | 20170419 | 5M |
| 5 | 20190301 | S5G | 20181119_2 | S5I | 20181218_4 | 5H | 20181210_2 | S5O | 20170426 | S5R |
| 6 | 20170419 | 5B | 20170419 | S5J | | | 20181213 | S5P | | |
| 7 | 20170420 | S5A | 20170426 | 5D | | | | | | |
| 8 | 20170425 | 5C | 20170427 | S5K | | | | | | |
| 9 | 20170426 | 5A | | | | | | | | |
| 10 | 20170427 | S5B | | | | | | | | |

**Table S4**: Results of statistical analysis of differences between the photoautotrophic (PL) and heterotrophic layer (HL) at various water contents (WHC; %) observed during microsensor measurements. Calculations were conducted for the mean (A) oxygen saturation, (B) pH, (C) nitrite, (D) nitrate, and (E) ammonium content. Statistical results were obtained with the Mann-Whitney U test (OriginLab Corporation, Northampton, Massachusetts, USA). Significant values ($p<0.05$) are written in bold letters.

| A | | | | |
|---|---|---|---|---|
| | WHC | U | z | p-value |
| PL vs. HL | 30 | 1184 | 1.78674 | 0.07398 |
| | 35 | 1483 | 1.28848 | 0.19758 |
| | 40 | 1219 | 1.56621 | 0.1173 |
| | 45 | 1544 | 1.42641 | 0.15375 |
| | 50 | 2069 | 1.60813 | 0.10781 |
| | 55 | 435 | 0.71938 | 0.47191 |
| | 60 | 1690 | 1.6766 | 0.09362 |
| | 65 | 1364 | -1.6476 | 0.09942 |
| | 70 | 1002 | 0.47285 | 0.63632 |
| | 75 | 1070 | 0.89088 | 0.37299 |
| | 80 | 1517 | 0.6919 | 0.489 |
| | 85 | 1024 | 0.42543 | 0.67052 |
| | 90 | 2555 | 3.29291 | **$9.916e^{-4}$** |
| | 95 | 305 | 0.99716 | 0.31869 |
| | 100 | 2531 | 2.57346 | **0.01007** |

| B | | | | |
|---|---|---|---|---|
| | WHC | U | z | p-value |
| PL vs. HL | 30 | 885.5 | 1.22461 | 0.22072 |
| | 35 | 428 | -1.03956 | 0.29854 |
| | 40 | 419.5 | -2.90215 | **0.00371** |
| | 45 | 544 | -1.85677 | 0.06334 |
| | 50 | 569.5 | -1.57076 | 0.11624 |
| | 55 | 130 | -1.18897 | 0.23445 |
| | 60 | 782.5 | -3.15989 | **0.00158** |
| | 65 | 521 | -2.16964 | **0.03003** |
| | 70 | 173 | -2.61445 | **0.00894** |
| | 75 | 516.5 | -0.25846 | 0.79606 |
| | 80 | 1583.5 | 0.90909 | 0.3633 |
| | 85 | 409.5 | 0.84394 | 0.3987 |
| | 90 | 903 | 0.76255 | 0.44573 |
| | 95 | 242.5 | 0.917 | 0.35914 |
| | 100 | 724.5 | -0.70072 | 0.48348 |

| C | | | | |
|---|---|---|---|---|
| | WHC | U | z | p-value |
| PL vs. HL | 30 | 412 | 1.2633 | 0.20648 |
| | 35 | 551 | 0.71031 | 0.47751 |
| | 40 | 465 | 2.00142 | **0.04535** |
| | 45 | 457 | 1.86743 | 0.06184 |
| | 50 | 483 | 2.47538 | **0.01331** |
| | 55 | 511 | 2.77184 | **0.00557** |
| | 60 | 443 | 1.33134 | 0.18308 |
| | 65 | 478 | 2.05464 | **0.03991** |
| | 70 | 484 | 2.15326 | **0.0313** |
| | 75 | 500 | 2.25117 | **0.02437** |
| | 80 | 575 | 3.28066 | **0.00104** |
| | 85 | 492 | 2.81135 | **0.00493** |
| | 90 | 387 | 0.55886 | 0.57626 |
| | 95 | 16 | 0.08452 | 0.93265 |
| | 100 | 483 | 1.67377 | 0.09418 |

| D | | | | |
|---|---|---|---|---|
| | WHC | U | z | p-value |
| PL vs. HL | 30 | 810 | 3.96919 | **$7.212e^{-5}$** |
| | 35 | 835 | 3.70229 | **$2.137e^{-4}$** |
| | 40 | 885 | 4.14425 | **$3.409e^{-5}$** |
| | 45 | 918 | 4.24184 | **$2.217e^{-5}$** |
| | 50 | 969 | 4.39562 | **$1.105e^{-5}$** |
| | 55 | 1100 | 5.66383 | **$1.480e^{-8}$** |
| | 60 | 587 | 3.4224 | **$6.207e^{-4}$** |
| | 65 | 903 | 3.31648 | **$9.116e^{-4}$** |
| | 70 | 955 | 4.24404 | **$2.195e^{-5}$** |
| | 75 | 890 | 3.29771 | **$9.748e^{-4}$** |
| | 80 | 843 | 2.80094 | **0.0051** |
| | 85 | 840 | 3.2402 | **0.00119** |
| | 90 | 952 | 3.58748 | **$3.339e^{-4}$** |
| | 95 | 297 | 1.38706 | 0.16542 |
| | 100 | 908 | 2.31399 | **0.02067** |

| E | | | | |
|---|---|---|---|---|
| | WHC | U | z | p-value |
| PL vs. HL | 30 | 89 | 0.38576 | 0.69968 |
| | 35 | 325 | 0.9493 | 0.34247 |
| | 40 | 222 | 1.36104 | 0.1735 |
| | 45 | 237 | 1.36736 | 0.17151 |
| | 50 | 376 | 1.42682 | 0.15363 |
| | 55 | 106 | 0.08383 | 0.93319 |
| | 60 | 113 | 0.58751 | 0.55686 |
| | 65 | 393 | 2.12258 | **0.03379** |
| | 70 | 222 | 1.15546 | 0.2479 |
| | 75 | 290 | 2.56935 | **0.01019** |
| | 80 | 315 | 3.22816 | **0.00125** |
| | 85 | 273 | 2.12136 | **0.03389** |
| | 90 | 375 | 1.40792 | 0.15916 |
| | 95 | 24 | 2.34743 | **0.0189** |
| | 100 | 418 | 2.22054 | **0.02638** |

# References

1. Büdel, B.; Darienko, T.; Deutschewitz, K.; Dojani, S.; Friedl, T.; Mohr, K. I.; Salisch, M.; Reisser, W.; Weber, B. Southern african biological soil crusts are ubiquitous and highly diverse in drylands, being restricted by rainfall frequency. *Microb Ecol* **2009,** *57* (2), 229-247.
2. Dougherty, E.; Lotufo, R., Hands-on morphological image processing. SPIE Press: Bellingham WA, USA: 2003.
3. Mele, G.; Nicolazzo, M.; Colandrea, M. Conmorph - 3D morphology and connectivity analysis of porous media. *Mendeley Data* **2021,** *V1, doi: 10.17632/6f36m26nx9.1*.
4. Otsu, N. A threshold selection method from gray-level histograms. *IEEE Trans Syst Man Cybern Syst* **1979,** *9* (1), 62-66.
5. Hoch, H. C.; Galvani, C. D.; Szarowski, D. H.; Turner, J. N. Two new fluorescent dyes applicable for visualization of fungal cell walls. *Mycologia* **2005,** *97* (3), 580-588.
6. Li, C. H.; Tam, P. K. S. An iterative algorithm for minimum cross entropy thresholding. *Pattern Recognit Lett* **1998,** *19* (8), 771-776.
7. van der Walt, S.; Schönberger, J. L.; Nunez-Iglesias, J.; Boulogne, F.; Warner, J. D.; Yager, N.; Gouillart, E.; Yu, T. scikit-image: image processing in Python. *PeerJ* **2014,** *2*, e453.
8. de Beer, D.; Schramm, A.; Santegoeds, C. M.; Kuhl, M. A nitrite microsensor for profiling environmental biofilms. *Appl Environ Microb* **1997,** *63* (3), 973-977.
9. de Beer, D.; Sweerts, J.-P. R. A. Measurement of nitrate gradients with an ion-selective microelectrode. *Anal Chim Acta* **1989,** *219*, 351-356.
10. de Beer, D.; Sweerts, J. P. R. A.; Heuvel, J. C. Microelectrode measurement of ammonium profiles in freshwater sediments. *FEMS Microbiol Lett* **1991,** *86* (1), 1-6.
11. de Beer, D.; van den Heuvel, J. C. Response of ammonium-selective microelectrodes based on the neutral carrier nonactin. *Talanta* **1988,** *35* (9), 728-730.
12. Revsbech, N. P.; Jørgensen, B. B., Microelectrodes: Their use in microbial ecology. In *Adv Microb Ecol*, Marshall, K. C., Ed. Springer US: Boston, MA, 1986; pp 293-352.
13. Kühl, M.; Revsbech, N., *Biogeochemical Microsensors for Boundary Layer Studies*. Oxford University Press: Oxford, 2001; p 180-210.
14. Munoz, J. L.; Deyhimi, F.; Coles, J. A. Silanization of glass in the making of ion-sensitive microelectrodes. *J Neurosci Methods* **1983,** *8* (3), 231-247.
15. Oswald, R.; Behrendt, T.; Ermel, M.; Wu, D.; Su, H.; Cheng, Y.; Breuninger, C.; Moravek, A.; Mougin, E.; Delon, C.; Loubet, B.; Pommerening-Roser, A.; Sorgel, M.; Poschl, U.; Hoffmann, T.; Andreae, M. O.; Meixner, F. X.; Trebs, I. HONO emissions from soil bacteria as a major source of atmospheric reactive nitrogen. *Science* **2013,** *341* (6151), 1233-1235.
16. Su, H.; Cheng, Y. F.; Oswald, R.; Behrendt, T.; Trebs, I.; Meixner, F. X.; Andreae, M. O.; Cheng, P.; Zhang, Y.; Poschl, U. Soil nitrite as a source of atmospheric HONO and OH radicals. *Science* **2011,** *333* (6049), 1616-1618.
17. Meusel, H.; Tamm, A.; Kuhn, U.; Wu, D.; Leifke, A. L.; Fiedler, S.; Ruckteschler, N.; Yordanova, P.; Lang-Yona, N.; Pöhlker, M.; Lelieveld, J.; Hoffmann, T.; Pöschl, U.; Su, H.; Weber, B.; Cheng, Y. Emission of nitrous acid from soil and biological soil crusts represents an important source of HONO in the remote atmosphere in Cyprus. *Atmos Chem Phys* **2018,** *18* (2), 799-813.
18. Oswald, R.; Ermel, M.; Hens, K.; Novelli, A.; Ouwersloot, H. G.; Paasonen, P.; Petäjä, T.; Sipilä, M.; Keronen, P.; Bäck, J.; Königstedt, R.; Hosaynali Beygi, Z.; Fischer, H.; Bohn, B.; Kubistin, D.; Harder, H.; Martinez, M.; Williams, J.; Hoffmann, T.; Trebs, I.; Sörgel, M. A comparison of HONO budgets for two measurement heights at a field station within the boreal forest in Finland. *Atmos Chem Phys* **2015,** *15* (2), 799-813.
19. Weber, B.; Wu, D.; Tamm, A.; Ruckteschler, N.; Rodríguez-Caballero, E.; Steinkamp, J.; Meusel, H.; Elbert, W.; Behrendt, T.; Sörgel, M.; Cheng, Y.; Crutzen, P. J.; Su, H.; Pöschl, U. Biological soil crusts accelerate the nitrogen cycle through large NO and HONO emissions in drylands. *Proc Natl Acad Sci U S A* **2015,** *112* (50), 15384-15389.
20. Heland, J.; Kleffmann, J.; Kurtenbach, R.; Wiesen, P. A new instrument to measure gaseous nitrous acid (HONO) in the atmosphere. *Environ Sci Technol* **2001,** *35* (15), 3207-3212.
21. Kleffmann, J.; Heland, J.; Kurtenbach, R.; Lorzer, J.; Wiesen, P. A new instrument (LOPAP) for the detection of nitrous acid (HONO). *Sci Pollut Res* **2002,** *9* (4), 48-54.
